# Prognosis of duodenal gangliocytic paraganglioma with lymph node metastasis: is follow-up >5 years required?

**DOI:** 10.1093/jscr/rjab159

**Published:** 2021-05-04

**Authors:** Natesh Yepuri, Gautam R Vanga, Rana Naous, Sudhir Pasham, Sravan Ponnekanti, Sudhakar Kinthala

**Affiliations:** 1 Department of Surgery, SUNY Upstate Medical University, Syracuse, NY, USA; 2 Department of Anesthesiology, Robert Packer Hospital, Sayre, PA, USA; 3 Department of Pathology, SUNY Upstate Medical University, Syracuse, NY, USA

## Abstract

Gangliocytic paragangliomas (GP) are rare tumors encountered exclusively in the second portion of the duodenum. Duodenal gangliocytic paraganglioma (DGP) belongs to a subclass of neuroendocrine neoplasms, characterized with unique histologic features of carcinoid tumor, paraganglioma and ganglioneuromas. According to the recent World Health Organization classification of gastrointestinal neuroendocrine tumors (NETs), there is a debate to classify them either as low-grade NETs or as an independent entity. There are a few reports of regional lymph node (LN) metastasis that could argue DGP as a true neoplasm. However, majority of them had a benign course, raising the question of whether long-term follow-up is required. We report a case of a retroperitoneal LN involvement by metastatic GP and additionally performed a systematic review of the literature to determine the optimal follow-up, since no guidelines exist for this rare entity.

## INTRODUCTION

Gangliocytic paragangliomas (GP) are rare tumors encountered exclusively in the second portion of the duodenum [1]. Duodenal gangliocytic paraganglioma (DGP) belongs to a subclass of neuroendocrine neoplasms, characterized with its unique histologic features of carcinoid tumor, paraganglioma and ganglioneuromas [2]. According to the recent World Health Organization classification of gastrointestinal (GI) NETs in 2010, there is a debate whether to classify them either as low-grade NETs or as an independent entity depending on their clinical behavior and prognosis [3]. There were few reported cases of regional lymph node metastasis that could argue DGP is a true neoplasm [4]. However, majority of tumors had a benign course during the follow-up, raising the question of long-term follow-up. We report a case of a retroperitoneal lymph node involved by metastatic GP and performed a review of the literature to determine the optimal follow-up of a DGP with lymph node metastasis, since no guidelines are currently available for this rare entity.

**Figure 1:**
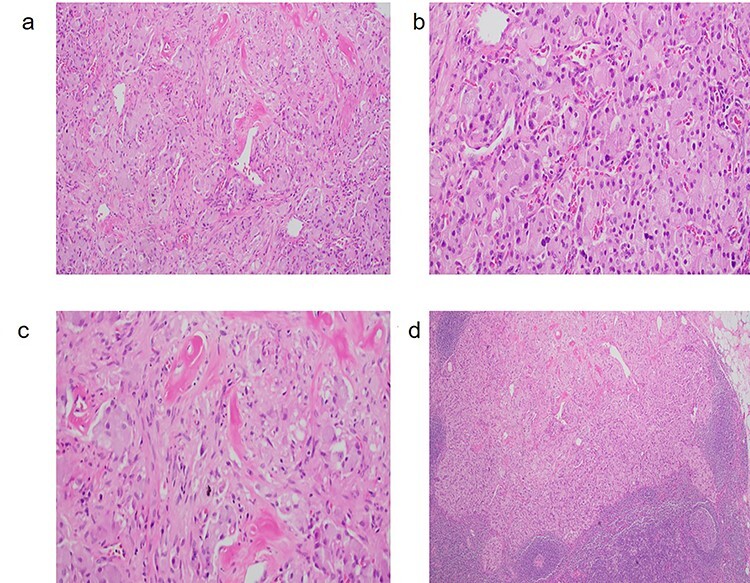
(**a**) GP with characteristic triphasic pattern: epithelioid endocrine-like cells admixed with an area of spindle Schwann-like cells and scattered ganglion type cells. (**b**) The epithelioid endocrine-like cells are arranged in compact nests reminiscent of carcinoid tumor or paraganglioma; (**c**) spindle Schwann-like cells are arranged haphazardly in a loose fascicular pattern. (**d**) GP tumor deposit, which metastasized to a regional lymph node.

**Figure 2:**
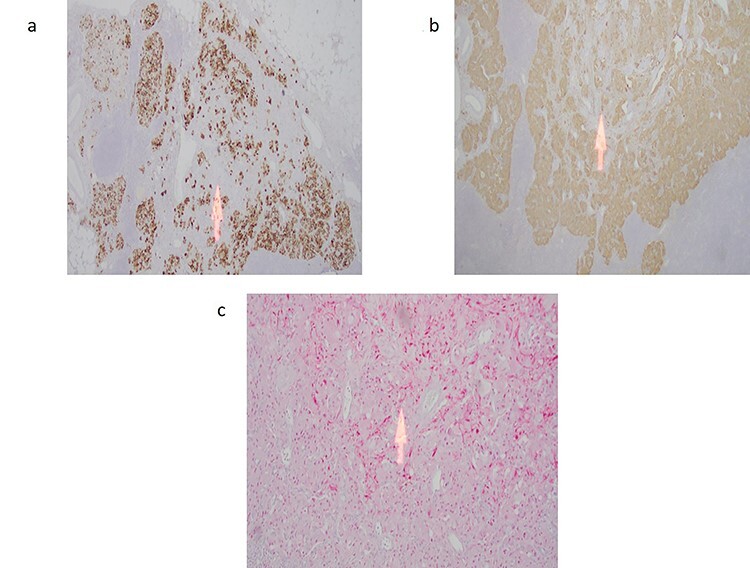
(**a**) Cytokeratin immunostain highlights the epithelioid endocrine-like cells with sparing of the spindle cell areas (arrow). (**b**) Synpatophysin immunostain illustrating cytoplasmic positivity in the epithelioid endocrine-like cells with absent staining in the spindle cell areas (arrow). (**c**) S100 immunostain highlights the spindle Schwann-like cells only (arrow) with negative staining in the epithelioid endocrine-like areas.

## CASE REPORT

A 59-year-old female was incidentally found to have a 2.5 cm mass in the descending portion of the duodenum near the ampulla, during her work up for shoulder pain. Endoscopic ultrasound (EUS) with fine needle aspiration (FNA) biopsy revealed atypical cells. Endoscopic retrograde cholangiopancreatography (ERCP) revealed both common bile duct and pancreatic duct dilation. Due to concern for malignant mass causing compression of the ampulla and common bile duct (CBD) ductal dilation, pancreaticoduodenectomy and lymph node dissection for excision of a periampullary and ampullary mass was done. Final pathology was consistent with GP. Microscopically, the tumor was present in the submucosa and invaded the lamina propria and muscularis propria. It was composed of three cell types: epithelioid cells, surrounding spindle-shaped sustentacular cells and scattered ganglion cells (Fig. 1a–c). Metastatic tumor deposits were seen in four out of six peripancreatic lymph nodes, with the largest metastasis being 1.3 cm, along with extra nodal extension. Immunohistochemical staining for synaptophysin, chromogranin, S100 and c-Kit was positive for duodenal tumor (Fig. 2a–c) and lymph node (Fig. 1d). In addition, the patient underwent treatment with external beam radiotherapy with intensity-modulated radiation therapy (IMRT) to a dose of 5040 cGy. At 5-year follow-up, she remained asymptomatic and without evidence of recurrence or metastatic disease.

**Figure 3:**
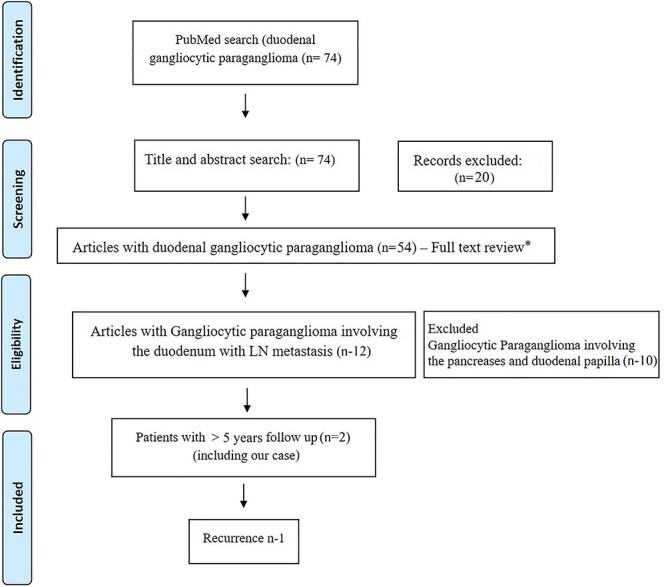
Prisma diagram showing systematic review of literature. * - excluded articles not relevant to duodenal gangliocytic paraganglioma (n-42).

## DISCUSSION

GPs are rare tumors with unclear origin, which predominantly affect patients in the third to ninth decade of life (median sixth decade). Though most often they occur sporadically, association with type I neurofibromatosis have been reported. These tumors are characterized by its triphasic cellular differentiation: epithelioid neuroendocrine cells, spindle cells with Schwann cell differentiation and ganglion cells [5]. These are usually solitary, submucosal nodules located in the second part of the duodenum with varying proportions of three populations of cells that includes endocrine, neuronal and peripheral nerve sheath elements [6].

They clinically manifest either in the form of abdominal pain, GI obstruction, and or as an incidental finding. Findings on ultrasonography include submucosal location of a hypoechoic or isoechoic lesion. On imaging with computed tomography and magnetic resonance imaging, these tumors are solid and homogeneous in appearance. Surgical resection remains the standard of care [7]. The use of adjuvant external radiotherapy was reported only in a single patient (besides us) that presented with locally advanced GP and lymph node metastasis [8]. GPs generally behave in a benign fashion, although cases of lymph node metastasis have been reported. Okubo *et al*. [7] reported that there is no significant difference in gender among patients with and without lymph node metastasis. However, significant difference in the rate of lymph node metastasis was reported in patients with DGP extending beyond the submucosal layer when compared with those confined to the submucosa.

In our review of literature of DGP with lymph node metastasis (Fig. 3), there has been only one recurrent case, whereas in majority of cases, even no recurrence has been reported [3]. Barret *et al*. [9] reported a longest followed up case (8 years) with no recurrence. Ours is the only second reported case that underwent post-resection radiotherapy and also the second longest followed up (>5 years) duodenal gangliocytic case with lymph node metastasis without recurrence.

## CONCLUSION

Our case report and review suggest that even with lymph node involvement, there is prolonged disease-free survival. In conclusion, the presence of lymph node metastases does not seem to influence the prognosis but rather the treatment modality. However, no consensus regarding optimal follow-up of DGP with lymph node metastasis exists and further data and reports relevant to these cases are necessary for the determination of the appropriate strategy and surveillance following surgical excision of these tumors.
